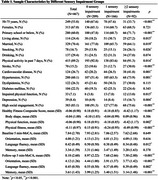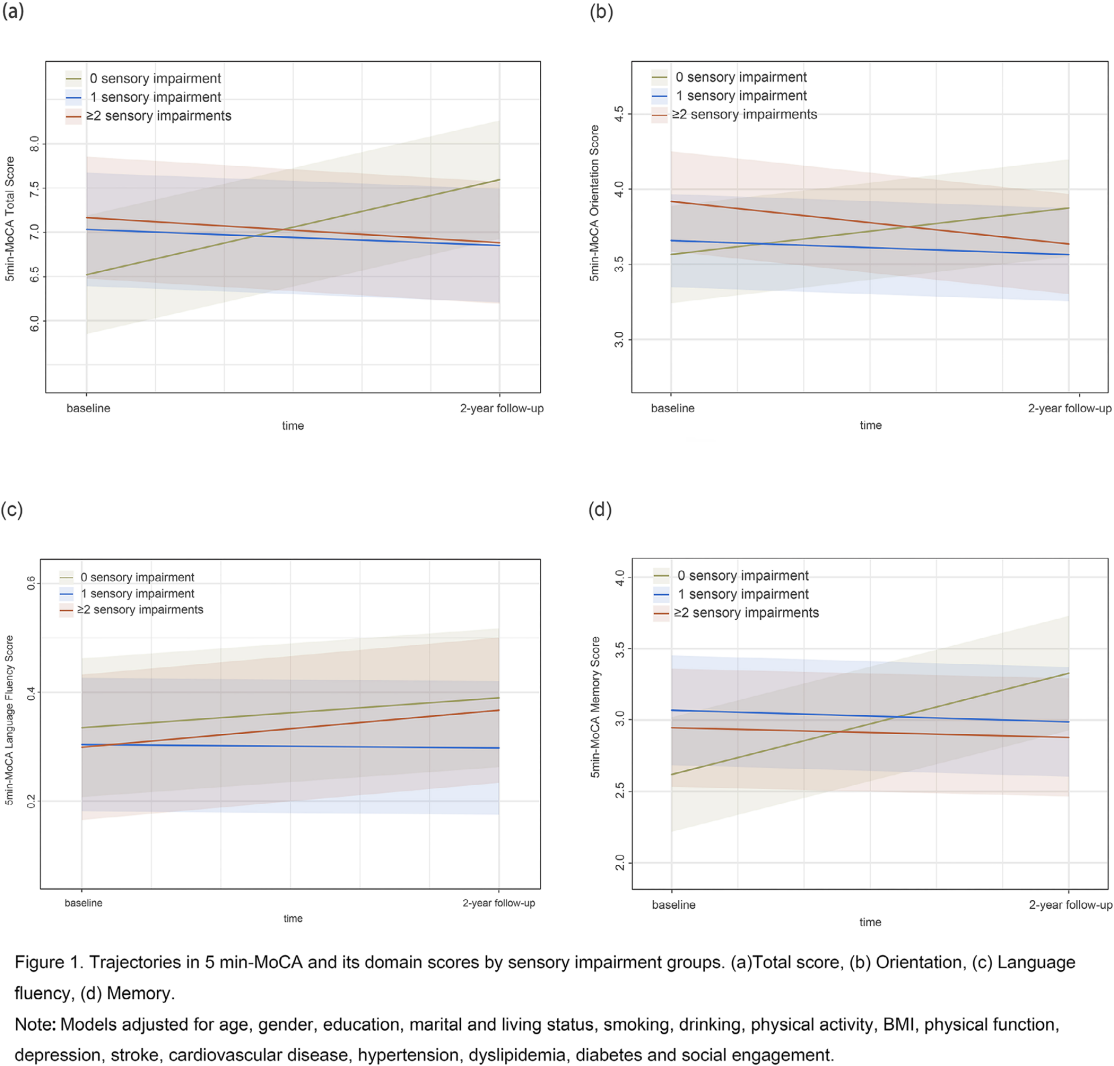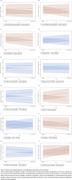# Multidimensional Health‐related Physical Fitness Moderates Multisensory Impairment and Cognitive Decline Among Dementia‐free Older Adults in China

**DOI:** 10.1002/alz70861_108519

**Published:** 2025-12-23

**Authors:** Xiaowen Lou, Ting Pang, Renwei Chen, Haoran Zhang, Xin Xu

**Affiliations:** ^1^ School of Public Health, the Second Affiliated Hospital of School of Medicine, Zhejiang University, Hangzhou, Zhejiang China; ^2^ Key Laboratory of Intelligent Preventive Medicine of Zhejiang Province, Hangzhou, Zhejiang China; ^3^ Memory, Ageing, and Cognition Centre (MACC), Department of Pharmacology, Yong Loo Lin School of Medicine, National University of Singapore, Singapore Singapore

## Abstract

**Background:**

Independent associations of visual, hearing and chewing functions with cognitive impairment have been demonstrated, whereas more evidence is needed for the cumulative effects of multisensory impairments. Although physical fitness is a modifiable factor in maintaining cognitive health, the specific dimensions modulate this cumulative effects remains to be addressed. Therefore, this study aimed to explore: 1) the longitudinal association between multisensory impairments and cognitive decline; 2) the moderating effect of various health‐related physical fitness dimensions in this association.

**Method:**

Eligible participants (age≥50 years, dementia‐free at baseline) were recruited from Chinese community. They completed demographic, cognitive, multisensory function, and health‐related fitness assessments at baseline and 2‐years follow‐up. Sensory impairments was categorized using self‐reported hearing, visual and chewing functioning (0, 1, ≥2 impairments). Cognitive function was measured by the 5 min‐Montreal Cognitive Assessment (5 min‐MoCA). Health‐related physical fitness was assessed using a composite score of 3 dimensions: body shape (BMI, waist circumference, waist‐to‐hip ratio), physical function (blood pressure, cardiorespiratory endurance), and physical fitness (gait speed, balance, flexibility, strength). Linear mixed‐effects models examined the longitudinal associations between multisensory impairments and cognitive decline, and the moderating effect of multidimensional health‐related physical fitness.

**Result:**

Of the 467 participants (mean age: 74.71±8.10 years, 67.0% female), 44.8% had no sensory impairment, 35.5% had single and 19.7% had ≥2 sensory impairments at baseline. During the 2‐year follow‐up, compared to the no‐impairment group, the single‐impairment group showed decreases in 5 min‐MoCA total score (*β*=‐1.26, *p*<0.001), orientation (*β*=‐0.40, *p*=0.002) and memory domains (*β*=‐0.79, *p*<0.001). The multiple‐impairment group exhibited greater reductions in 5‐min MoCA total (β=‐1.36, *p* <0.001) and orientation (β=‐0.59, *p* <0.001). Furthermore, overall health‐related physical fitness significantly moderated multisensory impairment and cognitive decline (*β*=0.68 *p*=0.025), with flexibility, strength, gait speed and balance also having moderating effects across cognitive domains (*p*<0.05).

**Conclusion:**

This study revealed the association of multiple sensory impairments with cognitive decline during 2 years and the moderating effect of multidimensional health‐related physical fitness indicators in Chinese dementia‐free elderly, which suggests the urgency of timely identification and management of sensory impairments in older adults, and encourages individuals to adopt enriched and targeted physical activities to reduce the risk of dementia.